# The Relationship Between Health Literacy Level and Metabolic Control Variables in People with Diabetes: A Cross-Sectional Study

**DOI:** 10.3390/healthcare14142149

**Published:** 2026-07-16

**Authors:** Nesibe Şimşekoğlu, Betül Erişmiş, Merve Feyza Demir Gürdal, Özge Pasin

**Affiliations:** 1 Fundamental of Nursing Department, Hamidiye Faculty of Nursing, University of Health Sciences, Istanbul 34668, Türkiye; 2 Department of Internal Medicine, University of Health Sciences Ankara Bilkent City Hospital, Ankara 06800, Türkiye; drbetulerismis@gmail.com; 3 Department of Internal Medicine, Bilecik Research Hospital, Bilecik 06800, Türkiye; mfeyzademiir@gmail.com; 4 Biostatistics Departmant, Hamidiye Medical Faculty, University of Health Sciences, İstanbul 34668, Türkiye; ozge.pasin@sbu.edu.tr

**Keywords:** diabetic, glucose metabolic disorders, health literacy, nursing

## Abstract

**Introduction:** Health literacy levels of individuals are decisive in the management of diabetes, which is characterized as a widespread public health problem. This study was designed to examine the relationship between health literacy level and metabolic control variables in people with diabetes. **Materials and Methods:** These descriptive cross-sectional studies data were collected using a questionnaire aimed to examine the demographic and metabolic variable characteristics of the participants and the Turkish Health Literacy Scale (THLS). Data were analyzed with appropriate statistical methods in SPSS (ver. 26) program. **Results:** The mean total score of the THLS was 29.74 ± 9.36. The health literacy levels of the participants were found to be ‘problematic/limited’. A significant difference was found between the participants’ mean score of THLS and the variables of ‘fasting blood glucose’, ‘educational status’, ‘social media use’, ‘internet use in physician selection’ and ‘looking for complaints online’. When evaluated using multivariate analyses, the effects of the independent variables on THLS were not found to be statistically significant. The model’s adjusted R^2^ value was 0.123. **Discussion and Conclusions:** People with diabetes, who constitute the sample of this study, cannot access sufficient information about the protection and improvement of their health levels, cannot interpret the information correctly and cannot transform it into behaviors. In diabetics with a limited health literacy level, it was determined that only the ‘Fasting blood sugar’ value, one of the metabolic control variables, had a negative relationship with the health literacy level.

## 1. Introduction

Diabetes is a physiological dysfunction characterized by hyperglycemia caused by deficiency or ineffective production of insulin in the pancreas. It may cause dysfunction in many body systems such as kidneys, eyes, heart and nerves, especially blood vessels [1,2]. The prevalence of diabetes is increasing rapidly with the increase in the world population, prolonged life expectancy, sedentary life and obesity. Diabetes leads to an increase in mortality and morbidity rate when left untreated [3,4].

Evidence from epidemiologic studies shows that, although there is a strong genetic basis for individual susceptibility to diabetes, many cases of diabetes can be prevented, or its development can be slowed, with early diagnosis in people with prediabetes, adequate treatment, and lifestyle changes. The main goal of diabetes management is to prevent the development of hyperglycemia and its complications by keeping blood glucose levels as close to normal as possible and to control risk factors. Treatment goals and methods should be planned specifically for each patient, taking individual risk factors into consideration [5,6]. Interventions to prevent the development of diabetes include intervention with antidiabetic and anti-obesity drugs as well as lifestyle behaviors such as lifestyle changes, education, exercise, diet and avoidance of smoking [6,7].

Health education, one of the multidisciplinary approaches to diabetes control, is based on cognitive ability and health literacy. The concept of health literacy is defined by the World Health Organization (WHO) as “the personal characteristics and social resources needed for individuals and communities to access, understand, evaluate and use information and services to make health decisions”. The WHO recognizes health literacy as an important component of achieving sustainable development goals. It is also reported to contribute to the achievement of health and social equity in societies [8,9]. It is accepted that the level of health literacy, in which nurses have a significant impact, is associated with health outcomes related to chronic diseases, including diabetes [10,11,12].

Studies in the literature show that low health literacy level, which is an important health outcome indicator, negatively affects diabetes self-management skills, glycemic and metabolic control levels. In addition, low health literacy level is associated with diabetes complications [10,11,13,14,15].

As diabetes and its complications have become a widespread public health problem, innovative approaches are needed to eliminate barriers in diabetes management. For these approaches to be successful, the health literacy levels of diabetic individuals are expected to be sufficient. Studies conducted with diabetic individuals indicate that diabetes self-care levels will increase with the improvement of health literacy and self-efficacy levels [16,17].

Nurses and physicians, who provide accessible healthcare services for sick individuals, play an important role in clinical training to increase health literacy levels. In particular, it has been determined that training given by diabetes nurses not only increases the level of health literacy but also increases patient satisfaction, supports self-management, and reduces diabetes complications and hospitalization times of patients [18,19]. In the national literature, while there are studies examining the level of health literacy in diabetic individuals [20,21], there are no studies examining its relationship with metabolic control values. It is thought that determining the health literacy levels of diabetic individuals and related metabolic factors will guide the diabetes education programs that are to be implemented by nurses and physicians. The aim of this study was to determine the level of health literacy in diabetic individuals and to examine its relationship with metabolic control variables.

In this context, answers to the following research questions were sought.

1. What is the level of health literacy among people with type 2 diabetes?
2. Is there an association between health literacy and metabolic control variables among people with type 2 diabetes?

## 2. Methods

### 2.1. Design

This study was designed as a descriptive cross-sectional study. The STROBE checklist for cross-sectional studies was used in reporting this study.

### 2.2. Place and Time of the Study

The study was conducted in the internal medicine outpatient clinic of a public hospital in Ankara between November 2023 and April 2024.

### 2.3. Study Setting and Sampling

The population of the study consisted of people with diabetes who applied to the internal medicine outpatient clinic of a state hospital in Ankara. Adequate sample size for the study was calculated using the G*power software (ver 3.1.9.7; Heinrich-Heine-Universität Düssseldorf, Düssseldorf, Germany). The required sample size for correlation analysis was calculated using an expected correlation coefficient of 0.50, a two-sided significance level of 0.05, and 80% statistical power. The minimum required sample size was determined to be 29 participants [22]. Of the 193 patients who met the inclusion criteria, 176 agreed to participate in the study, and the study was completed with 176 participants.

### 2.4. Inclusion Criteria

- Agreed to participate in the study by signing the informed consent form;
- Was diagnosed with diabetes at least 6 months ago;
- Over 18 years of age,
- Was examined in the internal medicine outpatient clinic of a public hospital in Ankara between November 2023 and April 2024;
- Has no visual or hearing impairment;
- Literate individuals will be included in the study.

### 2.5. Data Collection

The Introductory Information Form created by the researcher in line with the literature, the Turkish Health Literacy Scale (THLS-32) and an information form questioning the values of some metabolic variables were used as data collection tools.

#### 2.5.1. Introductory Information Form

The first 9 questions of the ‘Descriptive Information Form’, consisting of a total of 26 questions, assessed the socio-demographic characteristics of the participants. The next 9 questions examined disease-related characteristics, including self-reported treatment adherence. And the last 8 questions assessed computer and internet use.

#### 2.5.2. Türkiye Health Literacy Scale (THLS-32)

The THLS-32 scale was developed using the conceptual framework established in the “HLS-EU” study (HLS-EU Consortium, 2012) [23]. Turkish validity and reliability study was conducted by Okyay et al. (2016) [24]. It is a Likert-type scale consisting of thirty-two propositions and two sub-dimensions. Items 1–16 assess the dimensions of “treatment and service” and items 17–32 assess the dimensions of “disease prevention/health promotion”. Cronbach’s alpha coefficient for the Turkish reliability of the scale was found to be 0.927.

In the 5-point Likert-type scale, items are coded as “Very easy” 4 points, “Easy” 3 points, “Difficult” 2 points, “Very difficult” 1 point. “No opinion” option is defined as missing data. The total score obtained from the scale is calculated between 0 and 50. According to these scores, health literacy levels are divided into 4 basic groups.

“0–25 points”: “Insufficient”

“>25–33 points”: “Problematic-limited”

“>33–42 points”: “Adequate”

“>42–50 points”: “Excellent” [24].

#### 2.5.3. Metabolic Control Variables Information Form

The aim is to directly query metabolic control variables such as HbA1c, total cholesterol, HDL, LDL, triglycerides, microalbuminuria, urea, creatinine, weight, height, BMI, and systolic-diastolic blood pressure from hospital records.

The research data were collected by the researchers through face-to-face interviews, during which the participants completed the questionnaires in approximately 5 min. Laboratory results were extracted from the clinical database and corresponded to the same outpatient visit during which the questionnaires were completed.

### 2.6. Data Analysis

Descriptive statistics of quantitative variables in the study were given as mean, median, standard deviation, minimum and maximum, and descriptive statistics of qualitative variables were given in number and percentage form. The compatibility of quantitative variables with normal distribution was evaluated by a Kolmogorov–Smirnov test. The relationships between quantitative variables were evaluated by Spearman correlation analysis. A Mann–Whitney U test was used for mean comparison of two independent groups. A Kruskal–Wallis test was used for the mean comparison of more than two groups and Dunn’s test was used for multiple comparisons. Variables that were found to be statistically significant in the univariate analyses were included in the multiple linear regression model, from which the R^2^ and F statistics were obtained, and 95% confidence intervals were reported. Prior to the multiple linear regression analysis, the assumptions of the model were assessed. The standardized residuals were found to be approximately normally distributed, supporting the use of multiple linear regression. Statistical significance level was taken as 0.05 and the SPSS 26 (Released 2017. IBM SPSS Statistics for Windows, Version 26.0. Armonk, NY, USA: IBM Corp) package program was used for calculations. Participants with incomplete responses were excluded from the study as per the exclusion criteria. Therefore, no missing or indeterminate data were included in the final analysis.

## 3. Results

The findings regarding the socio-demographic and disease characteristics of the individuals included in the study are given in Table 1. The mean age of the participants was 55.15 ± 13.569, 97 (55.1%) were male, 132 (75%) were married, 86 (48.9%) were primary school graduates. It was determined that 52 (29.5%) of the participants were retired, 78 (44.3%) of them had an income equal to their expenses and 106 (60.2%) of them had other chronic diseases other than diabetes. When the data of the participants regarding diabetes disease were analyzed, 51 (29%) had a diabetes diagnosis period of 11–20 years, 49 (27.8%) went for diabetes control once a year, and 100 (56.8%) had a family history of diabetes. It was also determined that 89 (50.6%) of the participants evaluated their compliance with treatment as ‘moderate’, 81 (46%) of them complied with the diet, and 79 (44.9%) of them did not exercise regularly. When the internet usage characteristics of the participants were analyzed, the findings showed that 95 (54%) of them used the internet in choosing a hospital and a doctor, 158 (89.8%) did not use treatment other than the physician’s recommendation, and 163 (92.6%) did not discontinue the treatment recommended by the doctor due to the information learned on the internet. In addition, the results indicated that 91 (51.7%) of the participants did not do research about the disease on the internet before consulting a doctor, and 95 (54%) did not trust the information about health obtained from the internet.

The mean THLS total score of the participants, as shown in Table 2, was 29.7496 ± 9.36. The mean score of the ‘treatment and service’ sub-dimension of the THLS was 30.33 ± 9.8 and the mean score of the ‘disease prevention/health promotion’ sub-dimension was 29.45 ± 10.7. It was determined that the health literacy level of the participants was ‘problematic/limited’.

The relationship between the mean THLS total score and some metabolic control variables is given in Table 3. The results showed that the mean THLS total score had a statistically significant negative relationship only with fasting blood glucose (r = −0.145; *p* = 0.047). Although a statistically significant negative correlation was observed between health literacy and fasting blood glucose (Spearman’s rho = −0.145, *p* = 0.047), the effect size was small according to Cohen’s criteria. This suggests that the association has limited clinical relevance, and health literacy explains only a small proportion of the variability in fasting blood glucose. But when we performed a Benjamini–Hochberg false discovery rate (FDR) correction to account for multiple comparisons among the metabolic control variables, after FDR adjustment, the previously observed association between health literacy and fasting blood glucose was no longer statistically significant (FDR-adjusted *p* = 0.484). In addition, the findings indicated that the mean THLS score had no statistically significant relationship with HbA1c, total cholesterol, HDL, LDL, triglycerides, microalbumin, urea, creatinine, systolic and diastolic blood pressure (*p* > 0.05).

Some socio-demographic variables affecting the mean THLS total score of the participants are given in Table 4. And the linear regression results for THLS total score are given in Table 5. The Variance Inflation Factor (VIF) values for all independent variables were below 5. Statistically significant difference was observed between educational levels in terms of mean THLS scores (*p* = 0.046). When the differences were examined in detail, it was found that the mean THLS scores of those who were literate were statistically significantly lower than the mean scores of high school and undergraduate graduates (*p* = 0.035; *p* = 0.013). In addition, the mean THLS scores of primary school graduates were statistically significantly lower than the mean scores of undergraduate graduates (*p* = 0.025).

There was also a statistically significant difference between the mean THLS total score and the ‘treatment compliance’ variable (*p* = 0.042). When the differences were examined in detail, it was determined that the mean THLS scores of the participants with ‘good’ treatment compliance were higher than the mean scores of those with ‘poor’ treatment compliance (*p* = 0.049).

A statistically significant difference was observed between the THLS total mean score and the variable ‘Social media use status’ (*p* = 0.004). The analysis revealed that the mean THLS total score of the participants who used social media was significantly higher than those who did not use social media (*p* < 0.001).

The analysis revealed that there was a statistically significant difference between the THLS total mean score and the variable of ‘utilizing the internet in hospital and doctor selection’ (*p* = 0.036). The mean THLS total score of the participants who utilized the internet in hospital and doctor selection was significantly higher than those who did not (*p* = 0.022).

The results indicated that there was a statistically significant difference between the THLS total mean score and the variable ‘Research about the disease on the internet before consulting a doctor’ (*p* = 0.012). The THLS total mean scores of the participants who did research about the disease on the internet before consulting a doctor were significantly higher than those who did not (*p* = 0.003).

The overall multiple linear regression model was statistically significant (F = 5.835, *p* < 0.001), it explained only a modest proportion of the variance in health literacy (adjusted R^2^ = 0.123). After adjustment for the other variables included in the model, none of the individual predictors remained statistically significant. These findings suggest that the observed univariate associations were attenuated after controlling for potential confounding variables. The statistical significance of the overall regression model may, in part, reflect the number of dummy variables included in the analysis.

## 4. Discussion

The findings regarding the socio-demographic and disease characteristics of the individuals included in this study—which was designed to determine the level of health literacy in diabetic individuals and to examine its relationship with metabolic control variables—are given in Table 1. It was determined that the mean age of the participants included in the study was 55.15 ± 13.569, and the majority were male, married and had graduated from primary education. These findings support the information that diabetes is more common in male and middle-aged individuals [25].

In this study, the findings indicated that the participants were predominantly ‘retired’, had ‘income equal to expenses’ and had other chronic diseases other than diabetes. In the literature, it is discussed that individuals may experience financial difficulties considering the economic burden of managing diabetes and other diseases and this may negatively affect adherence to treatment [26,27]. In this context, it is considered important to financially support retired people with limited income.

When the data of the participants regarding diabetes disease are analyzed, it is seen that the majority were diagnosed with type 2 diabetes and the duration of diagnosis ranged between 11 and 20 years. The long duration of diabetes diagnosis reveals the importance of health literacy levels in diabetes management. In this study, the analysis revealed that although most of the participants had a family history of diabetes, they went to diabetes control only once a year, evaluated their compliance with treatment as “moderate”, and did not exercise regularly. This finding shows that individuals’ general health knowledge and awareness levels for diabetes management are not sufficient. In the literature, the importance of regular check-ups in diabetes management is emphasized; neglect behaviors in this regard are associated with low health literacy levels [28,29].

Although the findings obtained give the socio-demographic characteristics of the individuals constituting the sample of this study, there are studies in the literature that show similar sample characteristics, especially in terms of age, educational status, marital status and duration of diabetes [30,31,32]. In the study of Rachmawati et al. (2019), most of the participants were primary school graduates, similar to the results of this study, but differed in terms of gender [10]. The diversity of the findings obtained emphasizes the importance of interventions to increase individual awareness in diabetes management. In this context, it is recommended that health professionals should plan individualized education programs by considering the socio-demographic characteristics of individuals and use appropriate teaching methods.

When the internet usage characteristics of the participants included in this study were examined, the results showed that more than half of them used the internet to choose a hospital and a doctor, did not conduct research about the disease on the internet before applying to a doctor, and did not trust the information about health obtained from the internet. Most of the participants did not use treatment other than the physician’s recommendation and did not discontinue the treatment recommended by the doctor due to the information learned on the internet. These findings suggest that although individuals use the internet as a source of health information, they continue to rely on healthcare professionals when making health-related decisions. Consistent with these results, Jafari et al. (2023) reported that participants did not fully trust health information obtained from the internet and instead consulted healthcare professionals, particularly physicians and nurses, for health-related information [32].

In this study, the mean THLS total score of the participants was 29.7496 ± 9.36 (Table 2). The mean score of the ‘treatment and service’ sub-dimension of the THLS was 30.33 ± 9.8 and the mean score of the ‘disease prevention/health promotion’ sub-dimension was 29.45 ± 10.7. According to the findings obtained from the total score and sub-dimensions of the THLS, it was determined that the health literacy level of the participants was ‘problematic/limited’. For individuals to make the right choices and decisions for diabetes management, they need to have sufficient knowledge and health literacy [33]. The findings obtained from this research show that the individuals in the sample do not have access to sufficient information on the protection and improvement of their general health levels, especially diabetes, and cannot interpret the information correctly and may have difficulty in transforming it into behavior. Therefore, this suggests that the participants are inadequate in utilizing preventive health services, improving health, and managing treatment and rehabilitation processes. This situation also shows that the participants are not aware of their responsibilities regarding their own health and need professional support. In addition, the use of a general health literacy instrument (THLS-32) rather than a diabetes-specific health literacy scale may have limited the ability to assess diabetes-specific knowledge and self-management competencies, which could have influenced the observed associations.

In studies conducted with different sample groups in the literature, it has been reported that the level of health literacy is ‘limited’. This situation is associated with the participants’ level of education, inequalities in access to health services, and inadequacies in the methods of access to health information [18,24,34,35,36]. Inadequate health literacy may lead to an increased incidence of diabetes-related complications, especially in disadvantaged groups. When the literature on the subject is examined, the fact that studies conducted in different countries yield similar results reveals that inadequate health literacy is a global public health problem. This situation also creates an additional burden on the health systems of countries [37,38].

In the literature, recommended interventions to increase the level of health literacy include strengthening health communication, expanding community-based health education programs, and training health professionals on this issue. Studies have reported that high levels of health literacy positively support self-care behaviors, quality of life, and diabetes self-management skills of diabetic individuals [13,30,32,39,40]. In a study examining the relationship between health literacy levels and the successful aging status of elderly people with type 2 diabetes, unlike the results of this study, the health literacy levels of the participants were evaluated as ‘excellent’ [21]. In the study examining the effectiveness of an e-health guide to strengthen health literacy level, it was stated that the e-guide used increased the ability of patients to find and understand health information and thus increased their health literacy level. It was also stated that the greatest increase in health literacy level was seen in vulnerable patient groups [41].

In the randomized controlled trial of Goessl et al. (2019), it was reported that the diabetes prevention program using DVD to increase health literacy level was more effective than face-to-face sessions [34]. In another study, it was reported that the eHEALS application developed to increase the Digital Health Literacy level of parents of children with type 1 diabetes may affect the choice of treatment [20].

The relationship between the mean THLS total score and some metabolic control variables is given in Table 3. The results indicated that the mean THLS score only had a statistically significant negative relationship with fasting blood glucose. Although a statistically significant negative correlation was observed between health literacy and fasting blood glucose (Spearman’s rho= −0.145, *p* = 0.047), the effect size was small according to Cohen’s criteria. This suggests that the association has limited clinical relevance, and health literacy explains only a small proportion of the variability in fasting blood glucose. In addition, the results indicated that the mean THLS score had no statistically significant relationship with HbA1c, total cholesterol, HDL, LDL, triglycerides, microalbumin, urea, creatinine, systolic and diastolic blood pressure. This finding suggests a weak association between health literacy and fasting blood glucose rather than a direct effect. It suggests that individuals’ high health literacy level positively affects their health behaviors and, therefore, they may be more successful in managing their blood glucose levels. In the literature, there are studies reporting that high levels of health literacy have an improving effect on fasting blood glucose and other important metabolic control variables such as HbA1c and BMI [17,22,31,40,42]. Unlike HbA1c, which reflects average glycemic control over the previous 2–3 months, fasting blood glucose is more susceptible to short-term physiological and behavioral fluctuations. The observed association may reflect short-term variability rather than a stable relationship between health literacy and long-term glycemic control. In addition, there are findings that interventions focused on improving exercise, nutrition, self-efficacy perceptions and self-care behaviors also improve glycemic index value [14,22,30,33]. Since statistical significance does not always imply clinical significance, this finding should be interpreted with caution. Although health literacy is theoretically expected to contribute to improved metabolic control, our findings suggest that its direct association with metabolic outcomes may be limited. The weak association observed only for fasting blood glucose indicates that metabolic control is likely influenced by multiple interacting behavioral, psychosocial, and clinical factors. Therefore, health literacy should be considered an important, but not sufficient, determinant of diabetes outcomes. Furthermore, due to the cross-sectional design of the study, the direction of the observed association cannot be established. While higher health literacy may contribute to better glycemic control, it is also possible that individuals with better metabolic control have greater engagement with healthcare services and, consequently, higher health literacy. Longitudinal studies are needed to clarify the direction of this relationship.

The United Kingdom Prospective Diabetes Study (UKPDS) found that a 0.9% reduction in HbA1c was associated with a 25% reduction in the incidence of microvascular complications, a 10% reduction in diabetes-related mortality and a 6% reduction in all-cause mortality [43]. Low levels of health literacy have been associated with poor glycemic control, microvascular and macrovascular complications, especially retinopathy [22,35]. In a Burkina Faso-based study, it was concluded that there was a negative relationship between health literacy level and depression [44]. In addition, there are studies reporting that diabetes-related distress and depression negatively affect health literacy, self-efficacy and diabetes self-care behaviors [22,35,45,46]. In the meta-analysis study of Zheng (2018), it was reported that health literacy level was moderately associated with quality of life [7]. In the study of Tseng et al. (2017) based in Western Jamaica, it was suggested that health professionals should identify people with diabetes with low health literacy levels and take into account the difficulties faced by uneducated and elderly individuals [47]. In addition, the importance of strengthening the communication methods of health professionals to improve the self-management of people with diabetes was emphasized. Some studies have also indicated that all health professionals, especially physicians and nurses, should be trained in identifying and communicating with people with limited health literacy [47,48].

In a quasi-experimental study in which QR-coded information cards and mobile video methods were used to support the medication adherence and disease knowledge of people with low health literacy levels, the method used was reported to be effective [49]. In the literature, it has been reported that the health literacy level of parents with children diagnosed with diabetes is effective in diabetes control, especially glycemic control [20,50]. In a randomized controlled clinical trial based in Türkiye, the effect of health belief-based education on HbA1c in people with type 2 diabetes was examined. At the end of the study, it was stated that there was a decrease in the HbA1c value of the intervention group, as well as a significant improvement in self-efficacy, perceived sensitivity, perceived barrier and benefit [20].

According to the results of this study, it is noteworthy that health literacy level did not show a significant relationship with other metabolic control variables. This suggests that the relationship between health literacy level and some metabolic control variables cannot only be explained by the level of knowledge, and that situations such as the ability of patients to implement health behaviors and environmental factors may also affect this relationship. Studies have reported that social and cognitive factors such as sustainability of health behaviors, self-care behaviors, self-efficacy, social support, and access to health services, are also effective in diabetes management [40,42,51,52]. In studies examining the effect of health literacy level on cardiometabolic parameters in diabetic individuals, it was determined that the health literacy levels of the participants were associated with HbA1c, but not with blood pressure and LDL-C/K values [31]. Hansen D. et al. (2020) and Mbaezue N. et al. (2010) reported that health literacy level had no effect on self-monitoring of blood glucose [53,54]. In conclusion, the relationship between health literacy level and metabolic control variables is influenced by individual, environmental and behavioral factors. These results provide guidance in the planning of individualized education programs for diabetic individuals and interventions aiming to increase the level of health literacy.

Some socio-demographic variables affecting the mean THLS total score of the participants are given in Table 4. In the analysis, a statistically significant difference was observed between the educational status of the participants in terms of THLS total mean scores. Therefore, the mean THLS total scores of literate participants were found to be statistically significantly lower than the mean scores of high school and undergraduate graduates. This result supports the information that the level of education of individuals is an important determinant of their health literacy levels in line with the literature [44,55]. It is expected that people with a high level of education will also have adequate skills in accessing, making sense of and applying health information. This will positively affect individuals’ access to health services, compliance with treatment, and disease management. Especially in chronic diseases, the continuity of treatment compliance directly affects the health outcomes of individuals. The findings of this study reveal that people with a low education level should be considered as the primary target group in interventions to be planned to increase the level of health literacy. In the study of Jafari et al. (2023), in addition to the education level of the participants—unlike this study—gender, employment status and place of residence were also found to be effective on the level of health literacy [32]. It was reported that individuals who were employed, resided in the city, and were male had higher health literacy levels. Unlike the results of this study, there are studies that found significant differences in health literacy level in terms of age, gender, race, family history, economic status and time spent with diabetes [35,36,48,56].

A statistically significant difference was observed between the ‘treatment compliance’ variable in terms of THLS total mean scores. It was determined that the mean THLS scores of the participants with ‘good’ treatment adherence were higher than the mean scores of those with ‘poor’ treatment adherence. The findings of this study show that treatment adherence is closely related not only to individuals’ motivation or access to health services, but also to the ability to access, understand and apply health-related information. The result obtained supports the findings of the literature that health literacy level increases individuals’ ability to manage treatment processes effectively [55,57].

A statistically significant difference was observed in the THLS total mean scores according to social media use. Participants who used social media had significantly higher THLS total mean scores than those who did not. These findings suggest that social media use may positively influence health literacy. Social media provides individuals with opportunities to access health-related information and may support the development of health literacy. However, one of the major challenges in the management of chronic diseases such as diabetes is identifying reliable health information. Previous studies have questioned the reliability of health-related information shared on social media [58,59]. Educational interventions aimed at improving health literacy among individuals with diabetes should also focus on strengthening their ability to distinguish reliable sources from inaccurate or misleading online information.

A statistically significant difference was also observed according to participants’ use of the internet when selecting hospitals and physicians. Participants who reported using the internet for hospital and physician selection had significantly higher THLS total mean scores than those who did not. This finding suggests that internet use may contribute to higher health literacy by enabling individuals to make more informed healthcare decisions. Previous studies have reported that searching for information about hospitals and physicians online helps individuals make better-informed health decisions, although concerns regarding the reliability and security of online information remain [60,61].

Similarly, a statistically significant difference was found according to whether participants searched for information about their disease on the internet before consulting a physician. Participants who searched for disease-related information online before seeking medical care had significantly higher THLS total mean scores than those who did not. Advances in digital technologies have enabled individuals to access health information anytime and anywhere, increasing awareness of disease symptoms, treatment options, and potential risks. Consequently, patients may become better prepared for medical consultations and engage more actively in discussions regarding their treatment. Nevertheless, the accuracy and credibility of online health information should always be critically evaluated [59,61]. Even when patients obtain information from appropriate sources, the expected benefits may be limited if their health literacy levels are insufficient. Previous studies have shown that individuals tend to trust health information provided by physicians and nurses more than information obtained from the internet [44]. Furthermore, Jafari et al. (2023) and Güzel and Sağlam (2024) reported that seeking and verifying health information in digital environments positively affected health communication outcomes [32,62].

A limitation of this study is its limited sample representation. Since the study was conducted in a single outpatient clinic, selection bias cannot be excluded. Therefore, caution should be exercised when generalizing the findings to the wider population, as the results may not be representative of people with diabetes who are hospitalized, managed exclusively in primary care settings, or do not regularly attend outpatient follow-up. In addition, the cross-sectional design precludes any inference regarding causality or the direction of the observed associations. Therefore, reverse causality cannot be excluded, as individuals with better metabolic control may have greater engagement with healthcare services and, consequently, higher health literacy. Treatment adherence and health-information-seeking behaviors were assessed using self-reported data and may therefore be subject to recall and social desirability bias. Furthermore, the use of a general health literacy instrument (THLS-32) rather than a diabetes-specific health literacy scale may not have fully captured diabetes-specific knowledge and self-management competencies. To clarify the long-term relationship between health literacy and metabolic control variables, future multicenter studies involving repeated metabolic measurements, diabetes-specific health literacy assessments, and longitudinal designs are recommended.

## 5. Conclusions

In this study, which examined the level of health literacy among people with diabetes and its relationship with metabolic control variables, the participants were found to have a limited level of health literacy. In the bivariate analysis, a weak negative association was observed between health literacy and fasting blood glucose, whereas no significant associations were found with the other metabolic control variables. However, this association was not retained in the multivariable analysis, suggesting that the relationship should be interpreted with caution. Educational status, treatment adherence, social media use, using the internet to select a hospital or physician, and searching for disease-related information online before consulting a physician were associated with health literacy levels.

It is seen that there is a need for studies in which the level of health literacy and the metabolic control variables affected by it are examined in different sample groups with a larger sample number and different research methods. These studies are necessary to ensure the evidence-based development of interventions to increase the level of health literacy in an adequate and sustainable manner. Moreover, it is thought that the results to be obtained will guide physicians and nurses who assume an educational role in patient education programs for diabetes and guide the creation of higher education curricula.

## Figures and Tables

**Table 1:** Findings related to socio-demographic and disease characteristics of the participants (n = 176).

Variable		n (%)
Gender	Female	79 (44.9)
	Male	97 (55.1)
Marital status	Married	132 (75)
	Single	44 (25)
Educational background	Literate	9 (5.1)
	Primary education	86 (48.9)
	High school	42 (23.9)
	Bachelor’s degree	36 (20.5)
	Postgraduate	3 (1.7)
Profession	Housewife	62 (35.2)
	Employee	25 (14.2)
	Retired	52 (29.5)
	Other	37 (21.1)
Other chronic disease	No	70 (39.8)
	Yes	106 (60.2)
Diabetes diagnosis time	<1 year	13 (7.4)
	1–5 year	49 (27.8)
	6–10 year	48 (27.3)
	11–20 year	51 (29)
	>20 year	15 (8.5)
Diabetes control frequency	1 time per month	27 (15.4)
	1 time in 2 month	19 (10.8)
	1 time in 3 month	45 (25.6)
	1 time in 6 month	36 (20.5)
	1 time per year	49 (27.8)
Family history of diabetes	No	76 (43.2)
	Yes	100 (56.8)
Compliance with treatment	Good	65 (36.9)
	Middle	89 (50.6)
	Bad	22 (12.5)
Compliance with diet	Yes	81 (46)
	No	40 (22.7)
	Sometimes	55 (31.3)
Regular exercise	Yes	41 (23.3)
	No	79 (44.9)
	Sometimes	56 (31.8)
Using the social media when choosing a hospital and doctor	Yes	95 (54)
	No	66 (37.5)
	Sometimes	15 (8.5)
Research about the disease on the internet before consulting a doctor	Yes	62 (35.2)
	No	91 (51.7)
	Sometimes	23 (13.1)
Do not trust health information obtained from the internet	Yes	18 (10.2)
	No	95 (54)
	Not sure	63 (35.8)
Use of treatment other than physician recommendation	Yes	18 (10.2)
	No	158 (89.8)
Leaving the treatment recommended by the doctor due to information learned on the internet	Yes	7 (4)
	No	163 (92.6)
	Sometimes	6 (3.4)
Age	Mean + Sd	Med (Min–Max)
	55.15 ± 13.569	56 (18–88)

**Table 2:** THLS and subscale averages (n = 176).

	Mean ± Sd	Med (Min–Max)
THLS	29.7496 ± 9.36	30.72 (0–50)
Treatment and Service	30.33 ± 9.8	31.25 (0–50)
Disease prevention/Health promotion	29.45 ± 10.7	31.25 (0–50)

THLS Türkiye Health Literacy Scale.

**Table 3:** Association of THLS with metabolic control variables (n = 176).

Metabolic Control Variables	Mean ± Sd	Med (Min–Max)	THLS			FDR *p*
			r	*p*	%95 Confidence Interval	
Fasting blood sugar	149.42 ± 59	135 (68–371)	−0.145	0.047 *	−0.287 to 0.003	0.388
BMI	30.99 ± 8	29.38 (17.66–45)	−0.116	0.097	−0.260 to 0.032	0.388
HbA1c	8.164 ± 2.22	7.75 (5.2–14.8)	0.034	0.652	−0.114 to 0.180	0.823
Total cholesterol	186.39 ± 5.3	179.50 (89–292)	−0.004	0.961	−0.152 to 0.144	0.961
HDL	43.42 ± 14.7	42 (18–97)	0.078	0.302	−0.071 to 0.223	0.756
LDL	100.74 ± 34.2	96 (16–249)	0.057	0.454	−0.092 to 0.203	0.778
Triglyceride	224.60 ± 320	160.50 (53–648)	−0.059	0.434	−0.205 to 0.090	0.778
Microalbumin	29.13 ± 11.1	11.19 (0.32–44)	0.022	0.774	−0.126 to 0.169	0.844
Urea	37.179 ± 22.5	30.5 (12–128)	−0.076	0.088	−0.221 to 0.073	0.388
Creatinine	0.97 ± 0.56	0.86 (0.51–4.25)	−0.076	0.315	−0.221 to 0.073	0.756
Systolic blood pressure	137.30 ± 20.8	131 (90–202)	−0.031	0.686	−0.178 to 0.117	0.823
Diastolic blood pressure	80.02 ± 12.1	80 (53–145)	0.035	0.649	−0.113 to 0.181	0.823

THLS Türkiye Health Literacy Scale, * *p* < 0.05.

**Table 4:** Association of THLS with socio-demographic variables (n = 176).

		THLS				
Variable		Mean ± Sd	Med (Min–Max)	*p*	Post Hoc *p*	
Gender	Female	30.78 ± 9.10	32.29 (2–50)	0.168	-	
	Male	28.91 ± 9.54	29.69 (2–48)			
Marital status	Married	29.34 ± 8.72	30.73 (2–48)	0.444	-	
	Single	30.98 ± 11.09	31.25 (2–50)			
Educational background	Literate(1)	22.86 ± 22.86	25.52 (6–34)	0.046 *	1–2	0.172
	Primary education(2)	28.55 ± 10.95	29.17 (16–50)		1–3	0.035
					1–5	0.188
	High school(3)	31.14 ± 7.24	31.77 (16–48)		1–4	0.013
	Bachelor’s degree(4)	32.45 ± 5.59	33.07 (21–48)		2–3	0.118
					2–5	0.497
	Postgraduate(5)	32.99 ± 7.30	30.73 (27–41)		2–4	0.025
					3–5	0.860
					3–4	0.505
					5–4	0.938
Compliance with treatment	Good(1)	32.18 ± 9.07	32.81 (2–50)	0.042 *	1–2 = 0.27 1–3 = 0.049 2–3 = 0.599	
	Middle(2)	28.58 ± 8.88	29.69 (2–46)			
	Bad(3)	27.30 ± 10.92	28.91 (6–48)			
Using social media when choosing a hospital and doctor	Yes	31.82 ± 8.47	32.29 (2–50)	0.004 *	1–2 ≤ 0.001 1–3 = 0.163 2–3 = 0.490	
	No	26.64 ± 9.72	27.34 (2–48)			
	Sometimes	28.24 ± 10.38	29.43 (2–46)			
Using the internet when choosing a hospital and doctor	Yes	31.70 ± 8.44	32.29 (8–50)	0.036 *	1–2 = 0.022 1–3 = 0.092 2–3 = 0.723	
	No	27.83 ± 9.76	29.17 (2–46)			
	Sometimes	25.87 ± 10.77	28.13 (2–41)			
Research about the disease on the internet before consulting a doctor	Yes	32.56 ± 9.54	32.81 (2–50)	0.012 *	1–2 = 0.003 1–3 = 0.241 2–3 = 0.387	
	No	27.96 ± 9.54	29.17 (2–46)			
	Sometimes	29.26 ± 10.76	30.73 (2–48)			

THLS Türkiye Health Literacy Scale. * *p* < 0.05.

**Table 5:** Linear regression results for THLS.

		B	*p*	95% Confidence Interval for B	
				Lower Bound	Upper Bound
Educational background	Literate	−2.888	0.270	−8.038	2.261
	Primary education	1.792	0.258	−1.322	4.906
	High school	2.049	0.246	−1.421	5.519
	Bachelor’s degree	6.084	0.089	−.929	13.097
Compliance with treatment	Good	−1.589	0.251	−4.311	1.134
	Bad	−2.638	0.231	−6.969	1.693
Using the social media when choosing a hospital and doctor	No	−1.601	0.319	−4.766	1.563
	Sometimes	−.962	0.640	−5.012	3.088
Using the internet when choosing a hospital and doctor	No	−2.261	0.116	−5.088	0.565
	Sometimes	−2.312	0.274	−6.467	1.843
Research about the disease on the internet before consulting a doctor	Yes	2.186	0.166	−.912	5.285
	Sometimes	0.159	0.937	−3.808	4.126
Fasting blood sugar	-	−0.021	0.054	−0.042	0.000

F value: 5.835; *p* < 0.001; Adjusted R square: 0.123. In the multiple linear regression model, categorical variables were coded using dummy variables. The reference categories were defined as follows: Educational background: Literate; Compliance with treatment: Good; Using social media when choosing a hospital/doctor: Yes; Using the internet when choosing a hospital/doctor: Yes; Research about the disease on the internet before consulting a doctor: No.

## Data Availability

Access to the dataset is restricted due to ethical and privacy considerations related to survey participants. Although all data were anonymized prior to analysis, the dataset may still contain sensitive demographic and response information that could potentially allow participant identification when combined with other data sources. Therefore, the datasets used during the current study are available from the corresponding author upon reasonable request and subject to institutional and ethical considerations.

## Abbreviations

THLS	Turkish Health Literacy Scale
WHO	World Health Organization
HbA1c	Hemoglobin A1c
HDL	High-density lipoprotein
LDL	Low-density lipoprotein
C/K	Creatine Kinase
BMI	Body mass index
UKPDS	United Kingdom Prospective Diabetes Study
